# Determinants of cultural intelligence and cultural competence among undergraduate nursing students in Namibia: A multicampus study

**DOI:** 10.4102/hsag.v31i0.3159

**Published:** 2026-05-08

**Authors:** Nestor Tomas, Nangura H. Nyambe

**Affiliations:** 1 Department of General Nursing Science, Faculty of Health Sciences and Veterinary Medicine, University of Namibia, Rundu, Namibia

**Keywords:** cultural intelligence, cultural competence, patient-centred care, nursing education, cross-cultural care

## Abstract

**Background:**

In an increasingly globalised world, healthcare professionals encounter a diverse range of patients, making it essential for nursing students to be culturally intelligent and culturally competent. However, this area remains understudied in Namibia.

**Aim:**

To assess and describe the determinants of cultural intelligence (CQ) and cultural competence among undergraduate nursing students at two university campuses in Namibia.

**Setting:**

Two satellite university campuses in Namibia.

**Methods:**

A quantitative descriptive design with a purposive sample of 215 undergraduate nursing students was employed between May 2024 and July 2024 using the Cultural Intelligence Scale (CQS) and the Nursing Cultural Competence Scale (NCCS) online questionnaires. Data were analysed using SPSS v29.

**Results:**

Cultural competence had the highest average score (3.630 ± 1.150), followed by behavioural cultural intelligence (3.480 ± 1.010). A moderately significant correlation was observed between cultural competence and the behavioural dimension (*r* = 0.448; *p* < 0.001) and between motivation and the cognitive dimension (*r* = 0.310; *p* < 0.001). Four factors – behavioural cultural intelligence, cognitive, motivational and cultural competence – accounted for 47.62% to 61.17% of the total variance.

**Conclusion:**

Nursing students demonstrated strong capabilities in both the behavioural and motivational aspects of cultural intelligence, as well as in cultural competence. These findings are valuable for developing nursing curricula to train culturally intelligent and competent practitioners. Future studies should focus on tool refinement.

**Contribution:**

While refinement is required for the metacognitive subscale, the study contributes to the body of literature pertinent to curriculum design.

## Introduction

In light of the ever-expanding global community, there is a growing imperative to acknowledge the significance of developing cultural competence and cultural intelligence within the realm of nursing education (Brisbois & Pereira 2019). In this rapidly evolving landscape, the interplay between cultural intelligence and cultural competence has emerged as a crucial aspect of nursing education and practice. These two constructs are widely regarded as indispensable when it comes to providing care for diverse patient populations across a range of healthcare settings (Cruz et al. 2017; Farber 2019). Kruger et al. (2016) posited that the rise in interactions with patients from various cultural backgrounds and the growing global demand for cross-border healthcare work make it imperative for nurses to have the necessary cultural skills to cater for patients from diverse communities (Cruz et al. 2017).

The integration of cultural intelligence and cultural competence in nursing includes providing an environment of respect, dignity and inclusivity within the healthcare system (Parisa et al. 2016). Patients who feel that healthcare providers value their cultural identity are more likely to engage in their care and adhere to treatment plans, leading to improved health outcomes (Young & Guo 2020). Additionally, enhancing cultural competence and cultural intelligence in health professionals facilitates a shift from ethnocentric perspectives to ethno-relative orientations, which is a crucial progression in understanding and appreciating different cultures within their respective contexts (Li 2020). However, conflicts are increasingly becoming a major challenge to the provision of sensitive cultural care (Alkhaled et al. 2026). One such challenge is the mismatch between nurses’ and patients’ perceptions, communication difficulties and the need for self-determination and cultural norms (Majda et al. 2021a). Prior studies primarily examine cultural intelligence (Aboelenein & Mohamed 2022; Çelik & Yurdakul 2020; Göl & Erkin 2019) and cultural competence (Chun et al. 2010; Cruz et al. 2017; Osmancevic, Großschädl & Lohrmann 2023) as distinct constructs. More than ever, the increasingly alarming diverse globalised population calls for nurses to be culturally intelligent and culturally competent, as lacking in one aspect of the two constructs may negatively affect health service provision (Majda et al. 2021a).

Cultural intelligence (CQ) is the ability to adapt effectively to new cultural environments, which can make individuals more successful in diverse settings than those who lack this skill (Lee & Hong 2021; Thomas et al. 2015). In a world of increasing diversity, cultural intelligence has become a crucial skill for healthcare professionals. For example, a student nurse with cultural intelligence can understand and adapt to cultural differences, which in turn helps them communicate more effectively and build stronger relationships with patients from various backgrounds (Rahimaghaee & Mozdbar 2017). Essentially, nurses must have the necessary knowledge and skills related to cultural intelligence so that they can effectively provide quality and culturally sensitive nursing care to patients regardless of their cultural background (Cruz et al. 2017; Jeffrey 2016). Various Cultural Intelligence Scale (CQS) measurement scales exist (Ang et al. 2007; Lee & Hong 2021; Thomas et al. 2015). However, this study used the CQS developed by Ang et al. (2007) to assess an individual’s cultural intelligence. The CQS has been widely used in research to measure cultural intelligence across various contexts and populations. It has demonstrated good reliability and validity, making it a valuable tool for assessing cultural intelligence through its four dimensions: metacognitive, cognitive, motivational and behavioural CQ (Ang et al. 2007). Cultural competence is defined as care that is responsive to diverse patient populations and to cultural factors that can influence health, such as language, communication styles, beliefs, attitudes and behaviours (Cruz et al. 2017). Developing this competence helps reduce disparities in healthcare. In recent years, significant attention has been given to cultural competence among nurses and nursing students (Cruz et al. 2017), underscoring its paramount importance in the nursing profession. Key to developing this competence is displaying respect, sensitivity and a positive attitude towards patients, as all people want someone to care for them (Majda et al. 2021a).

Therefore, the subject of cultural competence and cultural intelligence is an important topic in the field of nursing education (Atalla & Elseesy 2023). Both of these major concepts are concerned with the provision of culturally sensitive nursing care to patients from diverse backgrounds. A common challenge in research is that these constructs are not always examined together in a single study. Additionally, concerns have been raised about whether the measurement tools used for them are consistent and valid across different cultures (Lee & Hong 2021). This study assessed and described the determinants of cultural intelligence and cultural competence among nursing students at two university campuses in Namibia. This research highlights the critical importance of both cultural intelligence and cultural competence in nursing education by examining the factors that influence their development in nursing students. It helps to close a knowledge gap regarding this topic in Namibia and provides valuable data on the validity of the tools used.

## Research methods and design

### Design

This study employed a quantitative descriptive cross-sectional design to capture numerical data and draw insights into the relationship between the constructs. This design has been used previously (An, Jin & Kim 2022; Li 2020) to investigate cultural intelligence and patient-centred care among nursing students.

### Setting

The study was conducted at two public university satellite campuses, namely, Rundu and Oshakati, in Namibia. These campuses offer nursing education to about 450 undergraduate students from diverse backgrounds and place their nursing students in local public and private health facilities. This unique context provides opportunities for students to develop and apply cultural intelligence and cultural competence before graduation. Moreover, the diverse student population with varying cultural backgrounds is essential for exploring the determinants of cultural intelligence and cultural competence in nursing practice (Senarathne & Meegoda 2021). The campuses were also accessible and convenient for the researchers, an important consideration during data collection.

### Population and sample

The target population consisted of 450 undergraduate senior nursing students from the third- to fourth-year level. A sample size was calculated using Slovin’s formula: N/(1 + N × a^^2^; Tejada & Punzalan 2012) using a 95% confidence level to select a sample of 223 nursing students through purposive sampling. To be eligible, participants had to be undergraduate nursing students from the third- to fourth-year level at the two university campuses in Namibia. The study excluded participants who were sick or unwilling to participate.

### Measures

The study adopted validated online questionnaires based on existing literature (Ang et al. 2007; Ross et al. 2010). The tools, that is, the CQS and the cultural competence scale, were readily available to the public and required no permission for use. The tools were piloted with 30 participants.

*Cultural Intelligence (CQ)*: The individual’s cultural intelligence was measured using the adopted CQS tool developed by Ang et al. (2007). The CQS has been widely used in research to measure cultural intelligence across various contexts and populations. The tool used 5-point Likert-type questions that rated responses from 1 = strongly disagree to 5 = strongly agree. A mean score of 3 or higher indicated a higher degree of CQ. The CQ consisted of 20 items across its four dimensions: metacognitive (4 items), cognitive (6 items), motivational (5 items) and behavioural CQ (5 items). The original tool demonstrated good reliability, with Cronbach’s alpha coefficients ranging from 0.77 to 0.84, making it a valuable tool for assessing cultural intelligence (Taber 2018).

*The Metacognitive* CQ dimension emphasised participants’ individual awareness and understanding of their own cultural assumptions and biases, as well as their ability to reflect on their cultural knowledge and adapt their thinking to various cultural contexts (e.g. ‘I am conscious of the cultural knowledge I use when interacting with people with different cultural backgrounds’ and ‘I am conscious of the cultural knowledge I apply to cross-cultural interaction’).

*The Cognitive* CQ dimension evaluated the participants’ knowledge and understanding of diverse cultural norms, values and practices, along with their ability to acquire and comprehend cultural information (e.g. ‘I know the rules of expressing nonverbal behaviours in other cultures’ and ‘I know the legal and economic systems of other cultures’).

The *Motivational* CQ dimension assessed participants’ interest, drive and confidence in engaging with people from various cultures, as well as their willingness to adapt their behaviour and learn from intercultural experiences (e.g. ‘I enjoy interacting with people from different cultures’ and ‘I am confident that I can socialise with locals in a culture that is unfamiliar to me’).

The *Behavioural* CQ dimension focused on participants’ ability to adapt both verbal and non-verbal behaviours to different cultural contexts, as well as their effectiveness in communicating and interacting with individuals from diverse cultural backgrounds (e.g. ‘I change my verbal behaviour [*e.g. accent, tone*] when cross-cultural interactions require it’ and ‘I use pauses and silence differently to suit different cross-cultural situations’).

*Cultural competence* was assessed using a tool adopted from Lin et al. (2019). The 19-item Nursing Cultural Competence Scale (NCCS) used 5-point Likert-type questions that rated responses from 1 = strongly disagree to 5 = strongly agree. A mean score of 3 or higher indicated a higher degree of cultural competence. The tool demonstrated comprehensive psychometric properties, with an acceptable Cronbach’s alpha of 0.91 (Barbera et al. 2020). In the current study, the Cronbach’s alpha of the NCCS was 0.94, indicating satisfactory internal consistency.

### Data collection

To give adequate time to collect and complete the online survey, data collection took place between May 2024 and August 2024 using a link that enabled participants to take part in the study. The researcher’s role was limited to distributing the questionnaire link via participants’ academic WhatsApp groups and sending up to two reminders. The survey was mobile-friendly, as it was distributed via WhatsApp. To participate, participants were requested to read the participant information leaflet and select the ‘agree’ button before proceeding with the rest of the survey questions. There was no clarification, explanation, questions or any other interference from the researcher during the data collection process, allowing participants to complete the survey in their preferred environments. The researcher sent a maximum of two periodic reminders to participants to encourage their involvement in the study. Furthermore, while participants were encouraged to participate in the study, no incentives were provided during data collection. Electronic materials collected were kept on the researcher’s personal computer, which is password-protected. Participants answered the questionnaires anonymously for about 10 min–20 min.

### Data analysis

Using SPSS Statistics version 29, data from 215 participants were analysed. This study followed classical test theory, using exploratory factor analysis (EFA) and Cronbach’s alpha as a framework to understand and improve the reliability of psychological tests. Exploratory factor analysis, specifically principal component analysis with varimax rotation and Kaiser normalisation, was conducted to identify underlying latent factors. The internal consistency of scales was evaluated through Cronbach’s alpha coefficients. Factor extraction was guided by the scree plot and the Kaiser–Meyer–Olkin Measure of Sampling Adequacy (KMO-MSA) criterion, with factors retained if their eigenvalues exceeded 1. As the tool used was adopted, a factor loading of at least 0.6 was considered acceptable (Awang 2014). Additionally, to ensure the robustness of the factor structure, each retained factor was required to explain at least 5% of the total variance and comprise a minimum of three variables (Baharum et al. 2023). Pearson correlation coefficients were computed to assess the relationships between study variables, with statistical significance set at *p* ≤ 0.05. Following Schober, Boer and Schwarte (2018), interpretations of correlation coefficients are as follows: 0.00–0.10 (negligible), 0.10–0.39 (weak), 0.40–0.69 (moderate), 0.70–0.89 (strong) and 0.90–1.00 (very strong).

### Ethical considerations

An application for full ethical approval was made to the Ethics Committee of the School of Nursing and Public Health at the University of Namibia, and ethics consent was received on 04 April 2024. The ethics approval number is SoN 26/2024. Before participation, all participants were thoroughly informed about the study’s objectives, procedures, potential risks and anticipated benefits. Written informed consent was obtained from all participants, signifying their voluntary agreement to participate. Participants were explicitly informed of their right to withdraw from the study at any point without incurring any penalty. The research was conducted in strict adherence to the ethical principles outlined in the Declaration of Helsinki.

## Results

### Participants’ demographic characteristics

Table 1 presents frequency statistics on the respondents’ gender, marital status, education level and faculty of study. Of a total of 215 (*n* = 215) participants, the largest majority share of 55% (*n* = 117) were in the age group 18–24 years, followed by 34% (*n* = 74) in the age group 25–30 years, while those in the age group 31–40 years accounted for the least proportion of 11% (*n* = 24). The distribution of respondents by marital status reveals that a disproportionate majority of 98% (*n* = 211) were single, while the remaining 2% (*n* = 4) were married. Participants in their fourth year accounted for 54% (*n* = 115) of the sample, and 46% (*n* = 100) were in their third year of study. In terms of campus of study, the majority, 69% (*n* = 148), were from the Oshakati campus, and 31% (*n* = 67) were from the Rundu campus.

**TABLE 1:** Demographic profiles of participants.

Variable	Frequency (*n*)	Proportion (%)
**Age group (years)**		
18–24	117	55
25–30	74	34
31–40	24	11
**Marital status**		
Single	211	98
Married	4	2
**Level of study**		
Third year	100	46
Fourth year	115	54
**Campus of study**		
Oshakati campus	148	69
Rundu campus	67	31

### Mean intelligence and competence

The mean scores for each domain of cultural intelligence among students were as follows: metacognitive dimension (3.250 ± 1.070), cognitive dimension (2.600 ± 1.050), motivational dimension (3.450 ± 1.050) and behavioural dimension (3.480 ± 1.010). Additionally, the mean score for the domain of cultural competence was recorded as 3.630 ± 1.150.

### Exploratory factor analysis

#### Sampling adequacy tests

As shown in Table 2, the KMO-MSA indicated that the sampling items for the ‘metacognitive’ sub-construct were inadequate, with a value of 0.511, which falls below the acceptable threshold of 0.6. Nonetheless, in this initial factor analysis, four items (e.g. MET01: ‘I am conscious of the cultural knowledge I use when interacting with people from different cultural backgrounds’; MET02: I adjust my cultural knowledge as I interact with people from cultures that are unfamiliar to me’; MET03: ‘I am conscious of the cultural knowledge I apply to cross-cultural interactions’; MET04: ‘I check the accuracy of my cultural knowledge as I interact with people from different cultures’) failed to load significantly on any dimension; hence, these three items were removed from further analysis.

**TABLE 2:** Kaiser–Meyer–Olkin measure of sampling adequacy and Bartlett’s test of sphericity statistics.

Constructs	No. of items	Correlation matrix determinant	KMO-MSA value	Bartlett’s test of sphericity χ^2^	*p*-value
Metacognitive CQ	3	0.996	0.511	895	0.827
Cognitive CQ	6	0.050	0.862	633.043	*p* < 0.001
Motivational CQ	5	0.096	0.814	495.826	*p* < 0.001
Behavioural CQ	5	0.061	0.837	589.779	*p* < 0.001
Nursing cultural competence	8	0.028	0.872	752.619	*p* < 0.001

KMO-MSA, Kaiser–Meyer–Olkin-measure of sampling adequacy; CQ, cultural intelligence; No., number.

Conversely, the cognitive, motivational and behavioural CQ sub-constructs, as well as the overarching ‘nursing cultural competence’ construct, demonstrated adequate sampling, with KMO-MSA scores exceeding 0.6. However, the correlation matrices for all four sub-constructs and the ‘nursing cultural competence’ construct were singular, suggesting that they could not be explained by linear combinations. Bartlett’s test of sphericity was employed to assess the factorability of the observed items. These findings confirm the suitability of the item data for factor analysis, supporting the application of EFA regarding explained total variances and the sizes and patterns of item loadings.

#### Total variance explained

The total variance explained results (Table 3) indicate that three sub-constructs of cultural intelligence had single factors extracted, with initial eigenvalues greater than one. The sub-construct ‘behavioural CQ’ accounted for the largest proportion of cumulative total variance explained, equal to 61.2%, while cognitive CQ and motivational CQ were almost equal at 55.3% and 55.4%, respectively. The cumulative total variance explained for the construct nursing cultural competence was marginally above 47.6%.

**TABLE 3:** Total variance explained.

Factor	Initial eigenvalues			Extraction sums of squared loadings		
	Total	% of variance	Cumulative %	Total	% of variance	Cumulative %
**Cognitive CQ**						
1	3.745	62.410	62.410	3.317	55.280	55.280
**Motivational CQ**						
1	3.195	63.893	63.893	2.770	55.408	55.408
**Behavioural CQ**						
1	3.424	68.487	68.487	3.058	61.167	61.167
**Nursing cultural competence**						
1	4.313	53.916	53.916	3.809	47.615	47.615

CQ, cultural intelligence.

### Factor structures

The factor loading results (Table 4) show that for the three sub-constructs measuring cultural intelligence, all items loaded meaningfully on single factors. For the cognitive CQ domain, participants reported knowing the following: cultural values and religious beliefs of other cultures (loading = 0.836), rules for expressing non-verbal behaviours in other cultures (loading = 0.798), the arts and crafts of other cultures (loading = 0.750), marriage systems of other cultures (loading = 0.742) and rules (e.g. vocabulary, grammar) of other languages (loading = 0.721).

**TABLE 4:** Factor structures.

Cognitive CQ	Factor 1
COG03. I know the cultural values and religious beliefs of other cultures.	0.836
COG06. I know the rules for expressing non-verbal behaviours in other cultures.	0.798
COG05. I know the arts and crafts of other cultures.	0.750
COG04. I know the marriage systems of other cultures.	0.742
COG02. I know the rules (e.g. vocabulary, grammar) of other languages.	0.721
COG01. I know the legal and economic systems of other cultures.	0.589
Extraction method: Alpha factoring.	-
a. One factor extracted. Five iterations required.	-
**Motivational CQ**	
MOT03. I am sure I can deal with the stress of adjusting to a culture that is new to me.	0.842
MOT02. I am confident that I can socialise with local people in a culture that is unfamiliar to me.	0.808
MOT04. I enjoy living in cultures that are unfamiliar to me.	0.753
MOT01. I enjoy interacting with people from different cultures.	0.659
MOT05. I am confident that I can get accustomed to the shopping conditions in a different culture.	0.637
Extraction method: Alpha factoring.	-
a. One factor extracted. Six iterations required.	-
**Behavioural CQ**	
BEH03. I vary the rate of my speech when a cross-cultural situation requires it.	0.850
BEH04. I change my non-verbal behaviour when a cross-cultural situation requires it.	0.847
BEH02. I use pauses and silence differently to suit different cross-cultural situations.	0.799
BEH01. I change my verbal behaviour (e.g. accent, tone) when a cross-cultural interaction requires it.	0.769
BEH05. I alter my facial expression when a cross-cultural interaction requires it.	0.624
Extraction method: Alpha factoring.	-
a. One factor extracted. Six iterations required.	-
**Nursing cultural competence**	
C03. I know that, clinically, individual cases or patients will think that the perineum is dirty.	0.791
C05. I know that, clinically, individual cases or patients will think of death as a taboo topic.	0.760
C04. I know that, clinically, individual cases or patients will believe that folk treatment is better than medical treatment.	0.756
C02. I know that, clinically, individual cases or patients will have their treatment affected by special cultural events.	0.720
C01. I know that, clinically, individual cases or patients will be concerned about homophonic issues.	0.681
C06. I know that, clinically, individual cases or patients will cause conflict in treatment because of different beliefs.	0.654
C19. I know that, clinically, individual cases or patients will reject treatment because of folk taboos.	0.641
Extraction method: Alpha factoring.	-
a. One factor extracted. Four iterations required.	-

CQ, cultural intelligence.

Regarding motivational CQ, respondents were confident that they could deal with the stress of adjusting to new cultures (loading = 0.842), socialise with local people in unfamiliar cultures (loading = 0.808), enjoy living in unfamiliar cultures (loading = 0.753), enjoy interacting with people from different cultures (loading = 0.659) and adapt to shopping conditions in different cultures (loading = 0.637). In relation to behavioural CQ, when cross-cultural interactions required it, participants varied their speaking rates (loading = 0.850), changed their non-verbal behaviour (loading = 0.847), used pauses and silence differently to suit diverse cross-cultural situations (loading = 0.799), altered their verbal behaviour (e.g. accent, tone; loading = 0.769) and changed their facial expressions (loading = 0.624).

Regarding nursing cultural competence, items C10, C12, C17 and C18 were eliminated after the first iteration, while C07, C08, C09, C11, C13, C14, C15 and C16 were removed after the second iteration, leading to only eight constructs loading successfully: thinking that the perineum is dirty (loading = 0.791), considering death a taboo topic (loading = 0.760), believing that folk treatment is better than medical treatment (loading = 0.756), treatment being affected because of special cultural events (loading = 0.720), being concerned about homophonic issues (loading = 0.681), conflict in treatment occurring because of diverse beliefs (loading = 0.654), rejecting treatment because of folk taboos (loading = 0.641) and self-learning when taking care of patients from different cultures (loading = 0.464).

### Scale reliability

Table 5 presents results indicating that Cronbach’s alpha coefficients exceed the minimum acceptable threshold (α = 0.7) for the internal consistency of items (Cronbach 1951). This confirms that the survey items for cultural CQ (cognitive CQ = 0.877; motivational CQ = 0.853; behavioural CQ = 0.882) and cultural competence (α = 0.871) effectively measure their respective unidimensional latent constructs.

**TABLE 5:** Scale reliability tests.

Constructs and items	Cronbach’s alpha	
	Item	Overall
**Cognitive CQ**		0.877
COG03. I know the cultural values and religious beliefs of other cultures.	0.877	-
COG06. I know the rules for expressing non-verbal behaviours in other cultures.	0.859	-
COG05. I know the arts and crafts of other cultures.	0.842	-
COG04. I know the marriage systems of other cultures.	0.855	-
COG02. I know the rules (e.g. vocabulary, grammar) of other languages.	0.854	-
COG01. I know the legal and economic systems of other cultures.	0.846	-
**Motivational CQ**		0.853
MOT03. I am sure I can deal with the stress of adjusting to a culture that is new to me.	0.837	-
MOT02. I am confident that I can socialise with local people in a culture that is unfamiliar to me.	0.807	-
MOT04. I enjoy living in cultures that are unfamiliar to me.	0.802	-
MOT01. I enjoy interacting with people from different cultures.	0.817	-
MOT05. I am confident that I can get accustomed to the shopping conditions in a different culture.	0.847	-
**Behavioural CQ**		0.882
BEH03. I vary the rate of my speech when a cross-cultural situation requires it.	0.856	-
BEH04. I change my non-verbal behaviour when a cross-cultural situation requires it.	0.852	-
BEH02. I use pauses and silence differently to suit different cross-cultural situations.	0.842	-
BEH01. I change my verbal behaviour (e.g. accent, tone) when a cross-cultural interaction requires it.	0.843	-
BEH05. I alter my facial expression when a cross-cultural interaction requires it.	0.887	-
**Nursing cultural competence**		0.871
C03. I know that, clinically, individual cases or patients will think that the perineum is dirty.	0.854	-
C05. I know that, clinically, individual cases or patients will think of death as a taboo topic.	0.851	-
C04. I know that, clinically, individual cases or patients will believe that folk treatment is better than medical treatment.	0.843	-
C02. I know that, clinically, individual cases or patients will have their treatment affected by special cultural events.	0.847	-
C01. I know that, clinically, individual cases or patients will be concerned about homophonic issues.	0.846	-
C06. I know that, clinically, individual cases or patients will cause conflict in treatment because of different beliefs.	0.857	-
C19. I know that, clinically, individual cases or patients will reject treatment because of folk taboos.	0.879	-
C16. When taking care of patients from different cultures, I will read books or watch medical television series for self-learning.	0.860	-

CQ, cultural intelligence.

### Correlations between constructs

Table 6 presents the correlation coefficient results, indicating that some constructs had significant moderate correlations, while others exhibited significant weak correlations. Cultural competence was significantly correlated with the cognitive (*r* = 0.214), motivational (*r* = 0.267) and behavioural (*r* = 0.448) dimensions. Behavioural competence was correlated with nursing metacognitive (*r* = 0.216), cognitive (*r* = 0.199) and motivational (*r* = 0.283) aspects, while motivation was correlated with the cognitive dimension (*r* = 0.312; *p* < 0.001).

**TABLE 6:** Pearson correlations (*N* = 215).

Correlations	Variables	Metacognitive	Cognitive	Motivational	Behavioural	Nursing cultural competence
Metacognitive CQ	Pearson correlation	1	-	-	-	-
	Sig. (2-tailed)	-	-	-	-	-
Cognitive CQ	Pearson correlation	0.068	1	-	-	-
	Sig. (2-tailed)	0.322	-	-	-	-
Motivational CQ	Pearson correlation	0.131	0.312**	1	-	-
	Sig. (2-tailed)	0.056	0.000	-	-	-
Behavioural CQ	Pearson correlation	0.216**	0.199**	0.283**	1	-
	Sig. (2-tailed)	0.001	0.003	0.000	-	-
Nursing cultural competence	Pearson correlation	0.026	0.214**	0.267**	0.448**	1
	Sig. (2-tailed)	0.706	0.002	0.000	0.000	-

CQ, cultural intelligence.

** , Correlation is significant at the 0.01 level (2-tailed).

## Discussion

The study investigated the determinants of cultural competence and cultural intelligence among nursing students at the Oshakati campus and the Rundu campus. The EFA revealed four factors that affect students’ cultural intelligence and cultural competence. Cognitive CQ was a major factor influencing cultural intelligence in this study. This finding demonstrates knowledge of other cultures, encompassing aspects such as values, beliefs, non-verbal behaviours, arts, marriage systems, languages and legal and economic systems (Robinson 2020). For example, students exhibited strong knowledge of cultural values and religious beliefs (loading = 0.836), rules for expressing non-verbal behaviours (loading = 0.798) and marriage systems (loading = 0.742). This strong knowledge can significantly benefit patients by enhancing communication, respecting family dynamics, increasing sensitivity to non-verbal cues and improving patient satisfaction (Majda et al. 2021a). As nursing students prepare to interact with patients from a variety of backgrounds, cognitive CQ – which entails knowledge of cultural norms, values and beliefs – is important. In a multicultural nation like Namibia, where medical personnel are faced with a complicated web of cultural viewpoints, this is of particular importance (Mlambo 2017). This finding is congruent with prior research by Presbitero (2016), which found cognitive CQ (β = 0.35, *p* < 0.05) to be a factor in measuring cultural intelligence in cross-cultural virtual communications. Cultural intelligence involves adapting to cultural differences and promoting effective communication in multicultural environments to deliver bias-free care (Rahimaghaee & Mozdbar 2017; Richard-Eaglin 2021). The cognitive domain of cultural intelligence encompasses not only the knowledge of cultural similarities and differences but also a deeper understanding of them (Richard-Eaglin 2021). According to Atalla and Elseesy (2023), there is a need for healthcare professionals to develop cultural intelligence to enhance their competency in cross-cultural interactions. Cultural intelligence is crucial for healthcare providers, especially nurses, to navigate diverse environments and mitigate decision-making biases that negatively affect health outcomes, ultimately improving equality in healthcare delivery (Richard-Eaglin 2021). Furthermore, nursing students with high cognitive and motivational CQ reflect their enthusiasm and preparedness to work in a multicultural setting, which is essential for delivering patient-centred care. The results of this study agree with research by Drossman et al. (2021), which highlights behavioural flexibility as an essential attribute for healthcare providers since it promotes rapport and trust with patients from various cultural backgrounds. This finding suggests that the students have acquired a broad and nuanced understanding of different cultural groups.

Motivational CQ significantly influenced nursing students’ cultural intelligence in this study, reflecting their confidence and eagerness to engage with diverse cultures (Pistorino 2020). It measures an individual’s drive and interest in adapting behaviour and learning from intercultural experiences (Van Dyne et al. 2012). Strengthening cultural intelligence training is crucial to enhance nursing students’ communication skills and confidence in providing culturally sensitive care (Chen et al. 2020; Üzar Özçetin & Sarıoğlu 2021). Similarly, behavioural CQ was a key determinant, reflecting the ability to adapt verbal and non-verbal behaviours for effective cross-cultural communication and interaction (Presbitero 2016).

While a recent study found a positive link between self-reported metacognitive CQ and other CQ subscales, its internal reliability and validity scores were unacceptably low. This contradicts the findings of a validated four-factor CQ structure proposed by Presbitero (2016). While earlier research using Ang et al.’s (2007) tool reported strong correlations among the four CQ dimensions, the tool has known limitations, including a lack of evidence for discriminant validity and measurement invariance across cultures (Lee & Hong 2021). This issue persists even though theory considers metacognition a core part of cultural intelligence (Goryunova 2025). This could be because of the limitations of classical test theory, as the analysis is highly dependent on the characteristics of the study participants (Am et al. 2023; Butakor 2022). Future research on metacognitive CQ should focus on improving the measurement tools.

This study reveals that nursing students possess a substantial understanding of culturally sensitive issues in healthcare, including patient views on death and perineal care. This finding is important, as cultural competence in nursing care is linked to increased patient satisfaction and improved health outcomes (Majda et al. 2021b). Prior research indicates that cultural competency enhances nursing students’ ability to deliver culturally sensitive care, reducing health disparities (Purnell 2018) and improving their attitudes towards cultural diversity and confidence in caring for diverse patients (Jones & Lee 2020). Therefore, nurses must be culturally mindful of individual client needs, adapting their practice to provide culturally safe and equitable care and employ effective cross-cultural communication (Antón-Solanas et al. 2021; Yari et al. 2020). While exposure to diverse languages and cultures is helpful, systematic and interactive approaches are more impactful in improving competence (Sharifi, Adib-Hajbaghery & Najafi 2019). Collectively, this evidence underscores the value of integrating cultural competency training into nursing curricula to prepare future nurses for diverse patient populations.

Pearson correlations revealed moderate to weak correlations between some of the factors, indicating the interconnectedness of these dimensions in shaping cultural competence. In this study, we found a positive and significant correlation between cognitive, behavioural and motivational CQ and cultural competence. These results partly agree with Atalla and Elseesy (2023), who found a statistically significant relationship between cultural intelligence and cultural competence. This suggests that changes in cultural intelligence may result in shifts in cultural competencies.

This study also found a significant but weaker correlation between motivational CQ and cognitive CQ (*r* = 0.312), suggesting that a positive attitude towards cultural diversity may motivate nurses to provide culturally sensitive care. This interconnectedness of the motivational and cognitive dimensions implies that a positive attitude towards cultural diversity can motivate nurses to deliver culturally sensitive care. While there is limited comparison of the relationships between cognitive CQ and motivational CQ involving nurses, a study on cultural intelligence in virtual communications by Presbitero (2016) found a significant correlation (*r* = 0.65, *p* ≤ 0.01). Nurses need the ability to apply their cultural knowledge and adapt their behaviours in clinical settings to provide culturally appropriate care. The practical value of understanding this relationship is that it can inform nursing education and practice to ensure graduates are equipped to deliver culturally competent care. Together, these constructs create a holistic approach to cross-cultural competence in nursing. While CQ emphasises adaptability and learning in diverse cultural contexts, cultural competence focuses on applying cultural understanding practically in patient care.

Clearly, with the exception of the metacognitive subscale, which requires further refinement, the three-structure model of CQ indicates that these subscales are significant factors in shaping students’ abilities. The three subscales (cognitive, motivational and behavioural) and cultural competence are fit for use in the Namibian context. Based on the study results, it is recommended for the University of Namibia to consider a training programme on cultural intelligence and competence to increase nursing students’ ability to interact, make judgment and demonstrate patience in the mid of managing patients from diverse cultural backgrounds.

### Strengths and limitations

With the exception of the metacognitive subscale, which requires further refinement, the three subscales of cultural intelligence (cognitive, motivational and behavioural CQ) and cultural competence can be utilised to assess these constructs among nursing students within the Namibian context. The findings are particularly pertinent to the design of nursing curricula aimed at training culturally competent nurses for both local and international markets. However, it is crucial to acknowledge certain limitations inherent in this research. The dependence on purposive sampling and self-reported data may introduce social desirability bias, necessitating caution when generalising the study results. Therefore, the researchers carefully recruited participants who met the study criteria to promote honest responses.

Furthermore, the lack of internal consistency and sampling adequacy in the metacognitive CQ domain indicates the need for refinement of the tool. Future research should prioritise the cross-validation of tools using sophisticated and contemporary theories, such as item response theory, to accurately measure the characteristics of each item.

## Conclusion

Nursing students showed strong capabilities in both the behavioural and motivational aspects of cultural intelligence. These findings suggest that having a positive attitude towards cultural diversity is a key motivator for nurses to provide sensitive and culturally competent care. Exploratory factor analysis revealed four key factors that shape nursing students’ cultural intelligence and competence: cognitive CQ, motivational CQ, behavioural CQ and nursing cultural competence. These findings are valuable for developing nursing curricula that prepare students to be culturally competent practitioners in both local and global healthcare settings. However, the invalid factor structure of the metacognitive CQ subscale suggests that the measurement tool requires refinement. Future research should prioritise tool cross-validation through confirmatory factor analysis, as well as assessing discriminant and convergent validity, to further substantiate the CQ tool’s inclusion in the design of the nursing curriculum.
